# Supplementary material for: The architecture and stabilisation of flagellotropic tailed bacteriophages
Source: Nat Commun. 2020 Jul 27;11:3748. doi: 10.1038/s41467-020-17505-w (PMC7385642; doi:10.1038/s41467-020-17505-w)
Supplement: Supplementary file 1 — Supplementary Information [file 41467_2020_17505_MOESM1_ESM.pdf]

SUPPLEMENT

The architecture and stabilisation of flagellotropic tailed bacteriophages

Joshua M. Hardy[1,+], Rhys A. Dunstan[2,+], Rhys Grinter[2], Matthew J. Belousoff[2], Jiawei Wang[2], Derek Pickard[3,4], Hariprasad Venugopal[5], Gordon Dougan[3,4], Trevor Lithgow[2,*] & Fasséli Coulibaly[1,*]

1. Infection & Immunity Program, Biomedicine Discovery Institute & Department of Biochemistry and Molecular Biology, Monash University, Clayton, VIC, Australia
2. Infection & Immunity Program, Biomedicine Discovery Institute & Department of Microbiology, Monash University, Clayton, VIC, Australia
3. Wellcome Trust Sanger Institute, Hinxton, Cambridge, CB10 1SA, United Kingdom
4. Department of Medicine, University of Cambridge, Addenbrooke's Hospital, Hills Road, Cambridge, UK.
5. Ramaciotti Centre for Cryo-Electron Microscopy, Monash University, Clayton, VIC, Australia

+ These authors contributed equally to the work

* To whom correspondence should be addressed: trevor.lithgow@monash.edu, fasseli.coulibaly@monash.edu

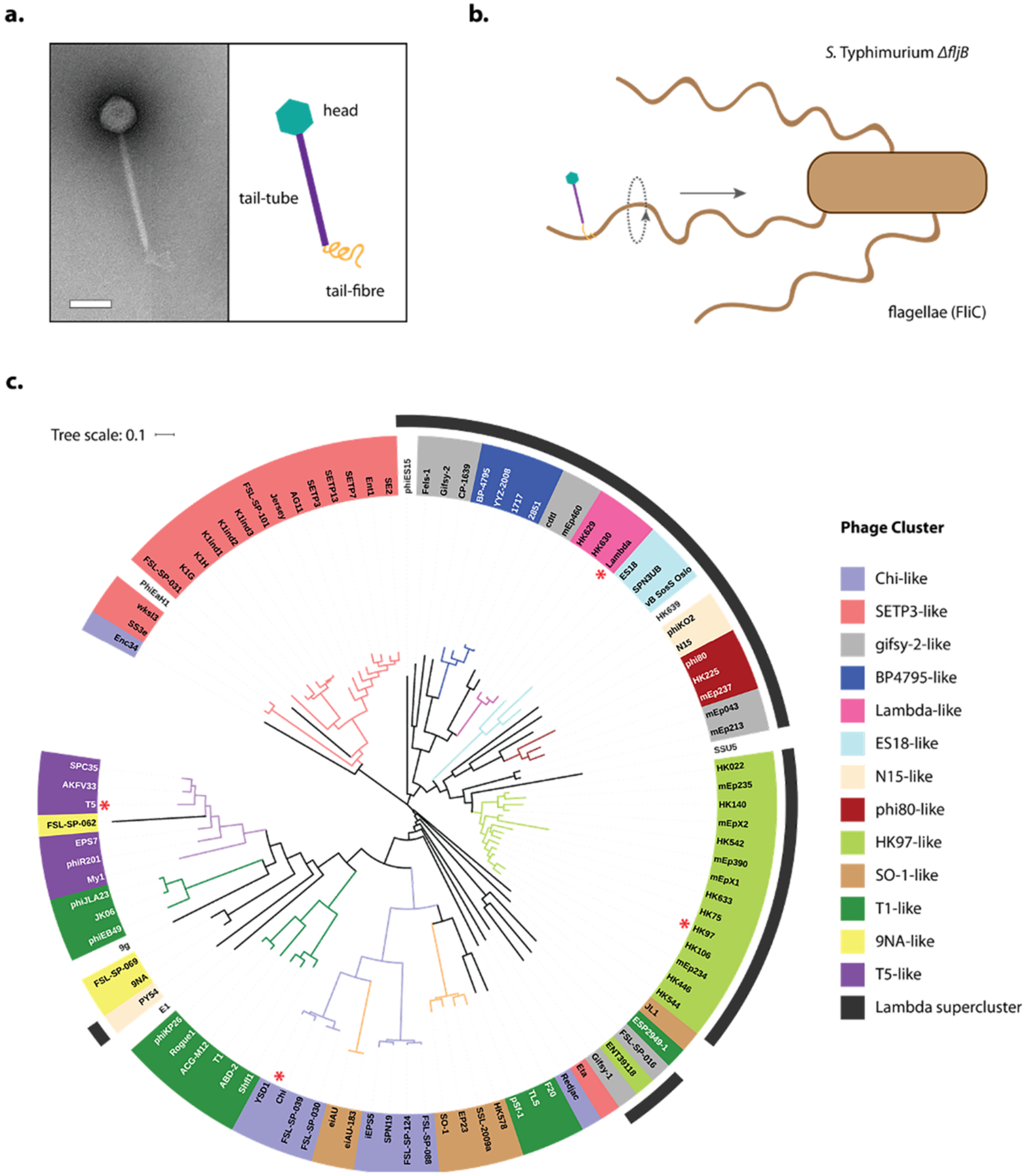

Supplementary Figure 1. Flagellotropic phage engage with host bacterial flagella in order to initiate infection. a. Negative stain image of a YSD1 particle (five imaging sessions), and cartoon representation of the same particle, indicating capsid, tail-tube and long tail-fibre. The scale bar represents 70 nm **b.** Salmonella have several flagella types, in S. Typhimurium (ΔfljB) all flagella are polymerized from FliC subunits, which are permissive for YSD1 binding. Interaction of the phage with the rotating flagella provides the basis for phage locomotion along the flagellum towards the Salmonella cell body, by a nut-on-bolt model of locomotion[1,2]. **c.** Tree based on the complete genome alignment of a highly-curated phage genome collection[3] (Supplementary Table 9). The color-coded text indicates the cluster nomenclature used by Grose and Casjens [3], including the archetypal phage for each cluster. The same cluster-specific coloring is used for the branches of the tree. YSD1 belongs to the χ-like group, which are highlighted in mauve. The red asterisks denote Chi and, because they are 97% sequence identical, YSD1, as well other viruses mentioned in the text.

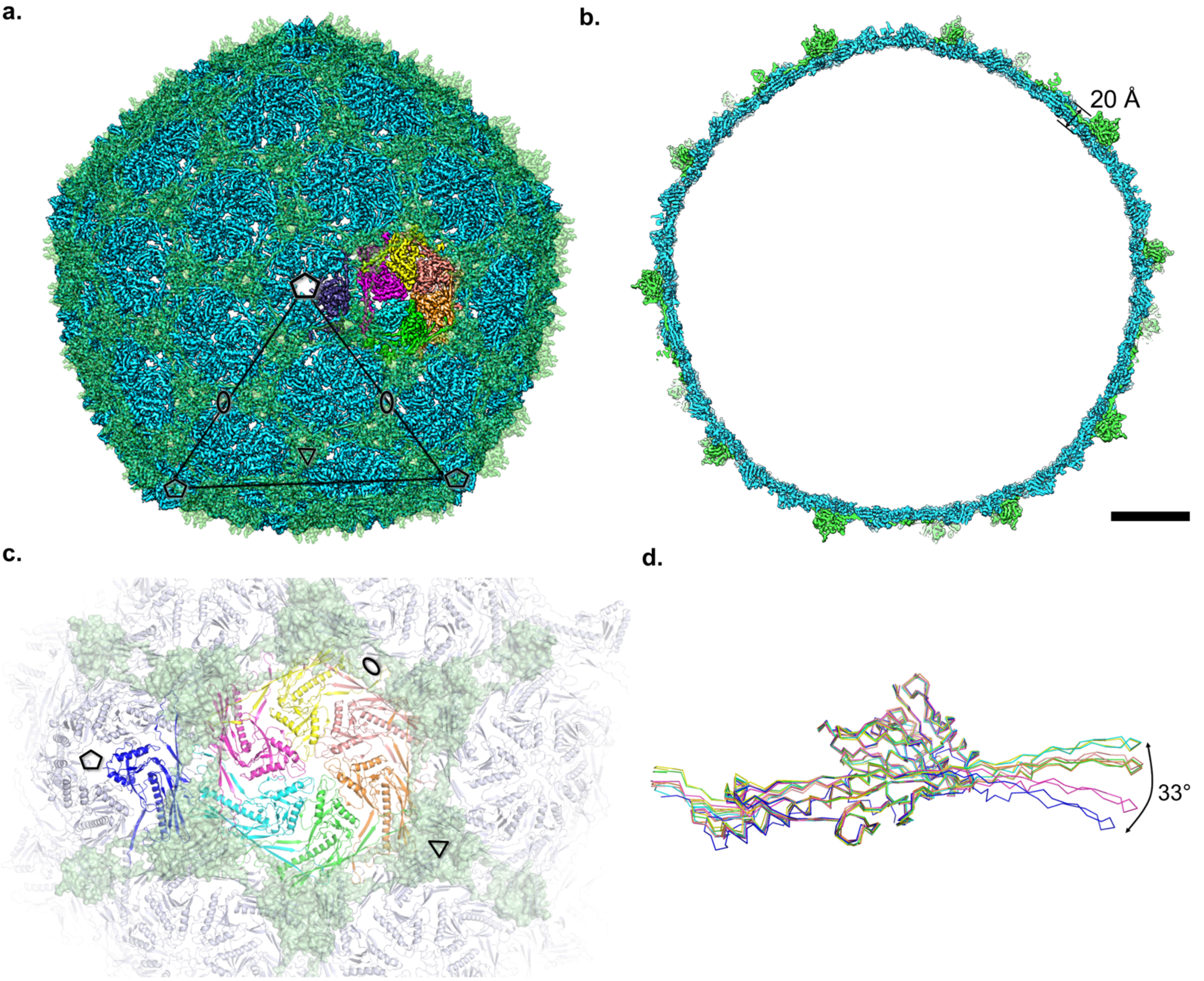

Supplementary Figure 2. The T=7l icosahedral YSD1 capsid. a. Surface representation of the MCP of YSD1 (blue) and auxiliary protein (green) with the icosahedral symmetry axes indicated. YSD1 has a T=7 laevo symmetry with six subunits forming a pseudo 6-fold symmetry axis and one pentameric subunit (dark blue) at the icosahedral 5-fold symmetry axis. **b.** Cross-sectional view of capsid with the average thickness measurement. Scale bar = 100 Å. **c.** Zoom of panel a on a cartoon depiction of the asymmetric unit of the MCP. The auxiliary protein is shown as a semi-transparent surface (pale green). **d.** Alignment of the chains from the asymmetric unit in PyMOL reveals the quasi-equivalent conformations of each subunit. E-loops that display the most pronounced bend are located at (blue) and around (magenta) the 5-fold vertex. Colours correspond to panel a and b.

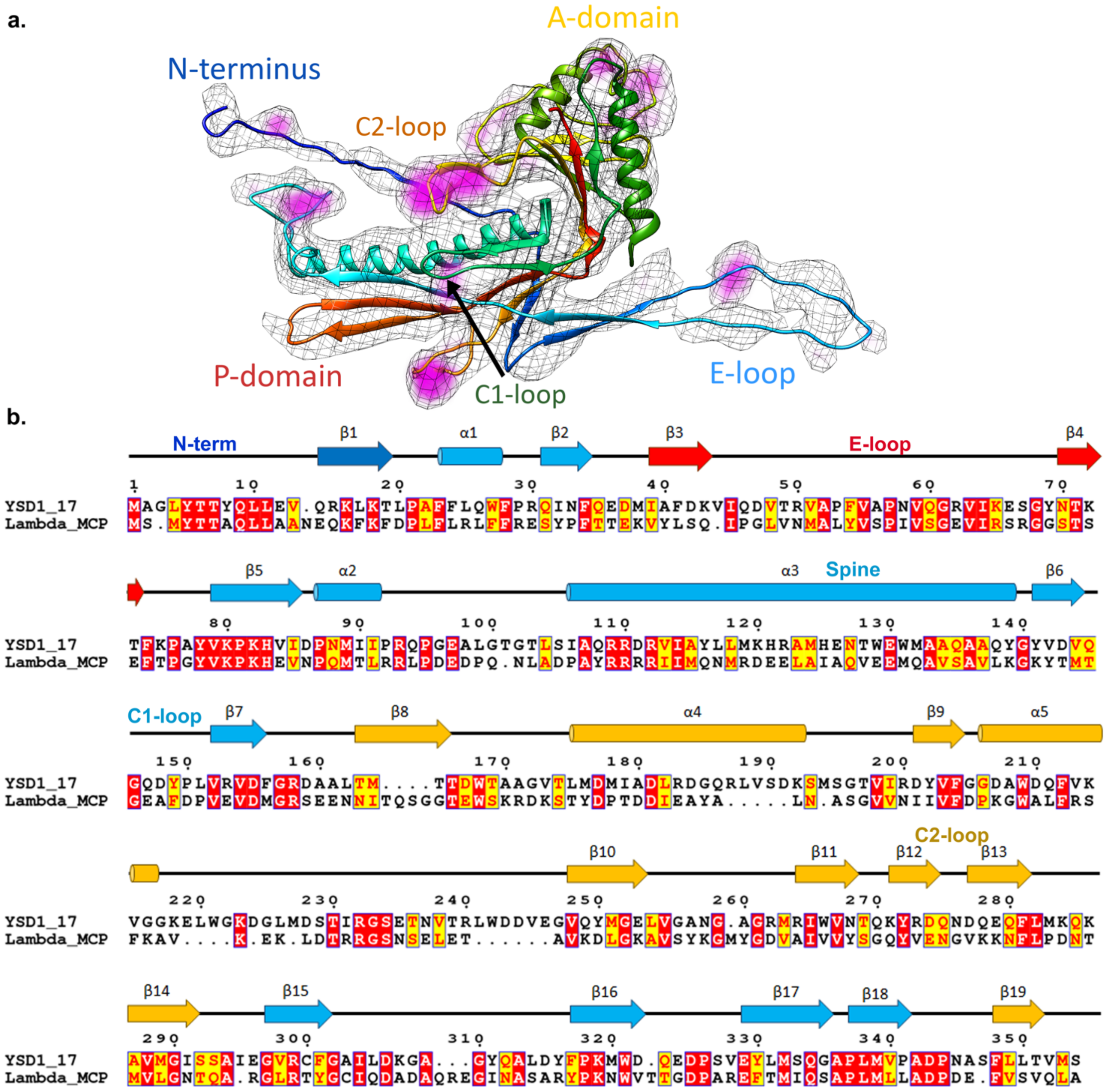

Supplementary Figure 3. The MCP of χ-like and λ phages are closely related and characterized by a clamp of loops C1 and C2. a. The MCP of YSD1 is depicted in a rainbow representation and annotated with structural features. A real-space fit of the MCP within the 6.8 Å cryo-EM reconstruction of λ phage capsid (EMD-5012, grey mesh) using Chimera[4] reveals a high level of similarity. The YSD1 MCP accounts for approximately 88 residues that HK97 gp5 (PDB:2FT1) does not account for (magenta). The A-domain of HK97 was fitted independently to the rest of HK97. **b.** Structure-based sequence alignment of the MCP of YSD1 and lambda. Beta-strands are displayed as arrows and alpha-helices as tubes. Secondary structure elements are colored by domain: N-terminus (blue), P-domain (cyan), E-loop (red), and A-domain (yellow). Sequence alignment was performed using T-COFFEE[5] and displayed using ESPRIPT[6]. Identical and similar residues are highlighted in red and yellow respectively.

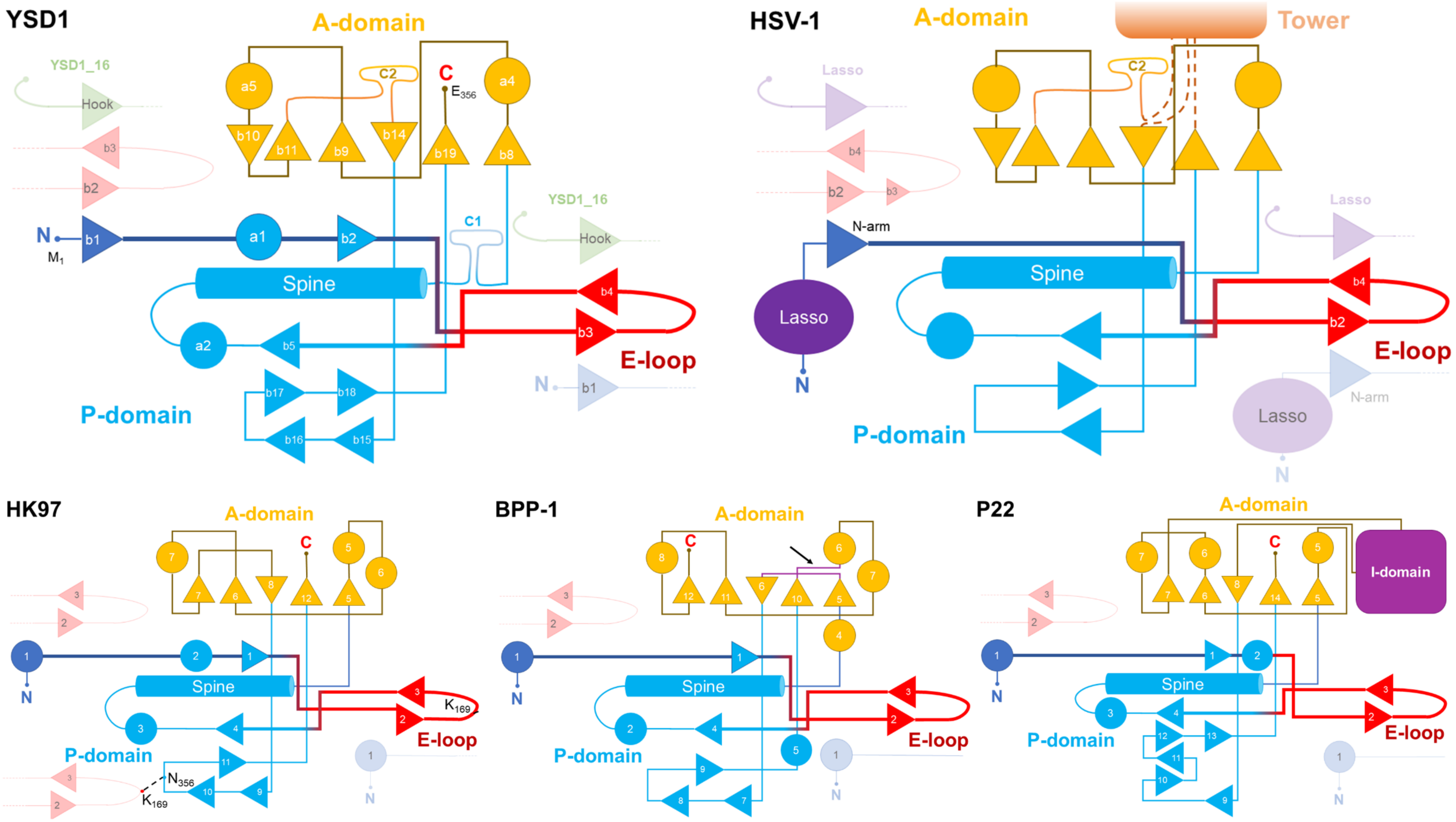

Supplementary Figure 4. The topology of the four classes of MCPs represented by the prototypical HK97, P22, BPP-1 and YSD1, and compared to a simplified topology of the HCMV herpesvirus. Augmented β-sheets from inter-molecular β-strands (shaded regions) are only indicated for YSD1 and HCMV with the colour scheme of **Figure 2**. The isopeptide bonds of HK97 and altered topology of BPP-1 are indicated.

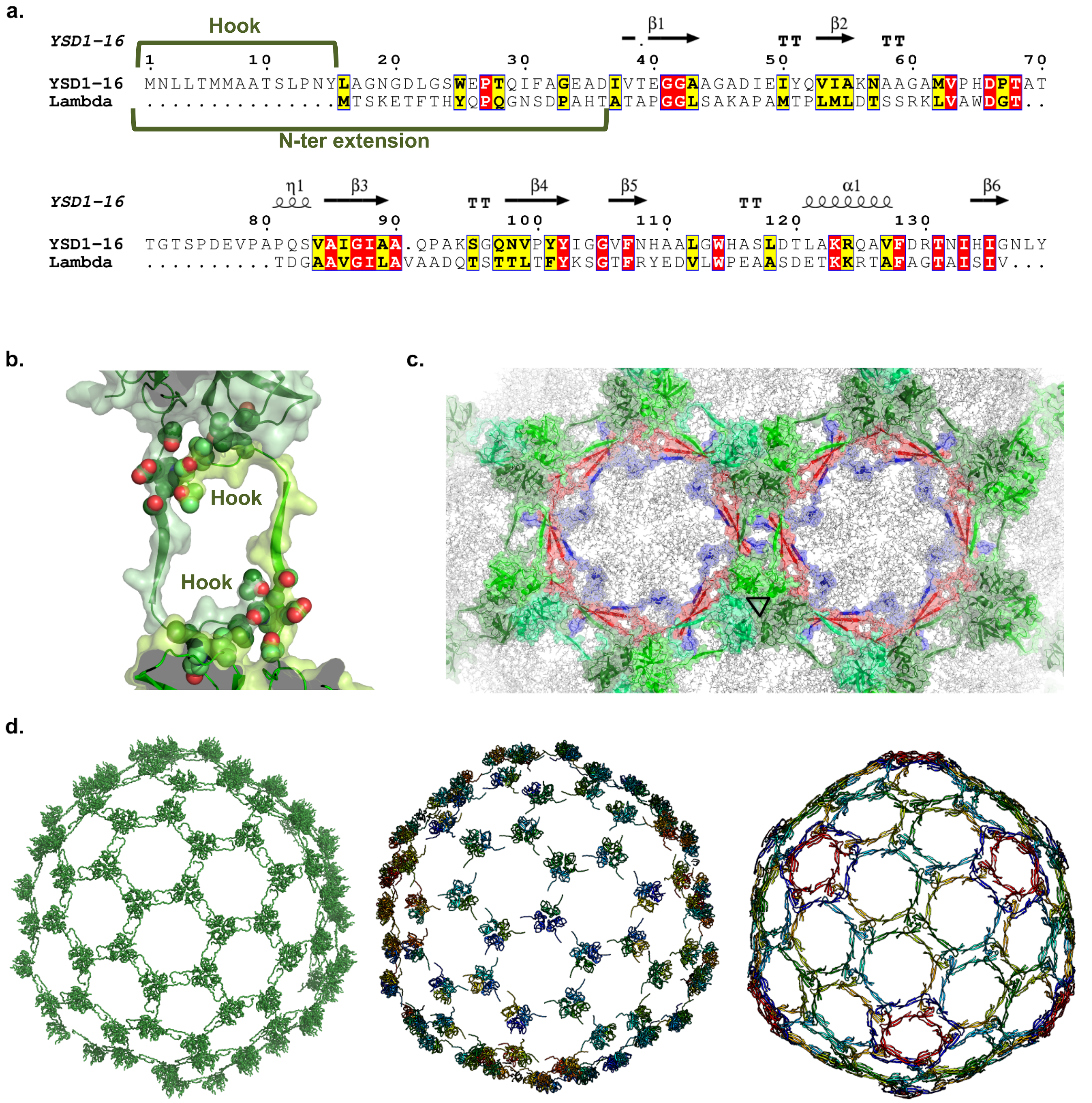

Supplementary Figure 5: A hook extension in auxiliary protein creates a distinctive outer chainmail in χ-like phages but not λ, which provides extra stabilisation. a. Structure-based sequence alignment of the auxiliary protein YSD1_16 and lambda gpD shows a high sequence conservation (17.4% identity) with an additional N-terminal sequence in YSD1 forming the so-called hook. Sequence alignment was performed using Clustal Omega[7] and displayed using ESPRIPT v3[6]. **b.** Two auxiliary trimers coloured in different shades of green are interconnected through the hook-shaped N-terminal extension. The side chains of interacting residues (inter-molecular distance < 5Å) are represented as spheres and coloured by element: green = carbon, red = oxygen, blue = nitrogen. **c.** Overview of the YSD1 outer chainmail formed by the auxiliary protein (green) and the MCP E-loops (red) and N-termini (blue). Colour scheme and orientation is similar to **Figure 2 b-d**. **d.** Comparison of siphovirus stabilisation networks displayed in a cartoon representation. YSD1 (left) combines two networks of interactions whereas the cementing protein (gpD) of lambda (middle) does not form connections between trimers. HK97 uses a covalent chainmail and no auxiliary protein (right).

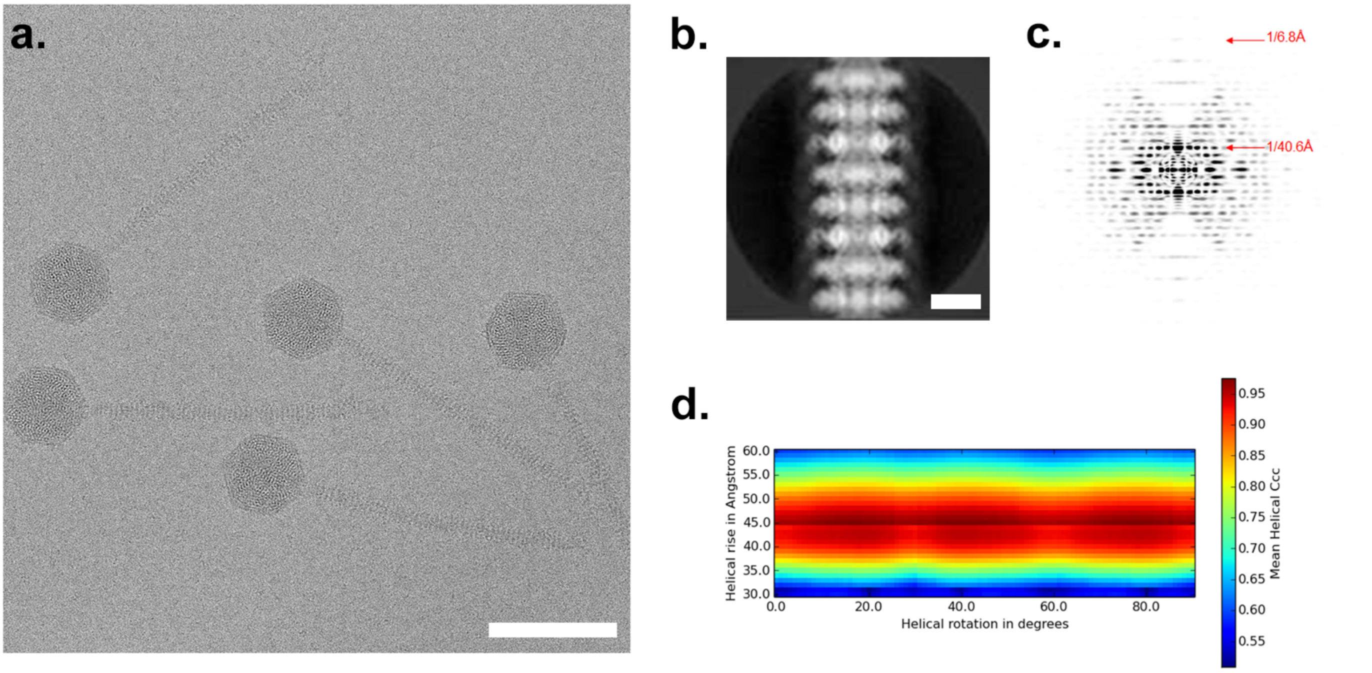

Supplementary Figure 6. Helical reconstruction of the YSD1 tail-tube. a. Representative cryo-EM micrograph of YSD1 (five imaging sessions). Scale bar = 100 nm. **b.** 2D class average of the YSD1 tail and **c.** the corresponding Fourier transform (inverted contrast). Domain 2 is seen as extensions from the tube and domain 3 is blurred at the edges. Two layer lines are labelled with the distance from the equator: the (n=0, 1/40.6 $Å^{-1}$) layer line corresponding to the helical rise and the highest resolution layer line visible (n=0, 1/6.8 $Å^{-1}$). **d.** Real-space search of the helical parameters using segclassreconstruct.py from the SPRING suite[8] around the approximate helical rise (30-60 Å) and 0-90° for the helical rotation. The mean cross-correlation of the projection of the reconstruction with the original 2D class is displayed as a heat map. Refinement of the parameters using RELION[9] indicated a helical rise of 40.61 Å and a twist of 19.69°.

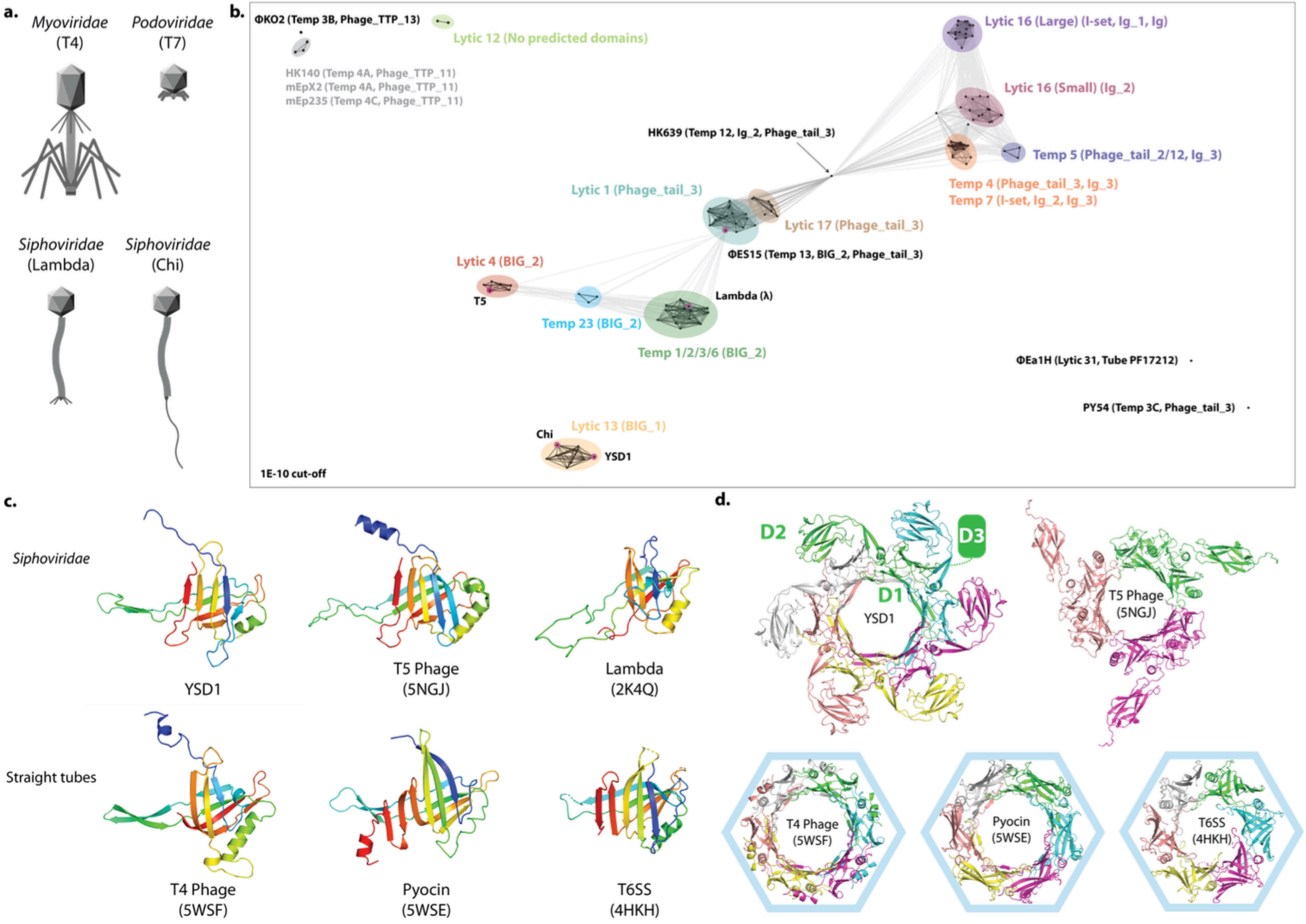
a.
Myoviridae
(T4)
Podoviridae
(T7)
Siphoviridae
(Lambda)
Siphoviridae
(Chi)
b.
ΦKO2 (Temp 3B, Phage_TTP_13)
Lytic 12 (No predicted domains)
HK140 (Temp 4A, Phage_TTP_11)
mEpX2 (Temp 4A, Phage_TTP_11)
mEp235 (Temp 4C, Phage_TTP_11)
Lytic 16 (Large) (I-set, Ig_1, Ig)
Lytic 16 (Small) (Ig_2)
HK639 (Temp 12, Ig_2, Phage_tail_3)
Temp 5 (Phage_tail_2/12, Ig_3)
Temp 4 (Phage_tail_3, Ig_3)
Temp 7 (I-set, Ig_2, Ig_3)
Lytic 1 (Phage_tail_3)
Lytic 17 (Phage_tail_3)
ΦES15 (Temp 13, BIG_2, Phage_tail_3)
Lytic 4 (BIG_2)
T5
Lambda (λ)
Temp 23 (BIG_2)
Temp 1/2/3/6 (BIG_2)
ΦEa1H (Lytic 31, Tube PF17212)
PY54 (Temp 3C, Phage_tail_3)
Chi
Lytic 13 (BIG_1)
YSD1
1E-10 cut-off
c.
Siphoviridae
YSD1
T5 Phage
(5NGJ)
Lambda
(2K4Q)
Straight tubes
T4 Phage
(5WSF)
Pyocin
(5WSE)
T6SS
(4HKH)
d.
D2
D3
D1
YSD1
T5 Phage
(5NGJ)
T4 Phage
(5WSF)
Pyocin
(5WSE)
T6SS
(4HKH)

Supplementary Figure 7. Tail morphology and tail-tube proteins show diversity within the *Siphoviridae*. a. Morphology of myophages (e.g. T4), podophages (e.g. T7) and siphophages (e.g. λ and YSD1). **b.** Sequence-based relationships for TTPs is documented with CLANS[10], graphically depicting sequence-based homology in large protein sequence datasets. The analysis utilizes all-against-all pairwise BLAST to cluster representations (black dots) of individual protein sequences in three-dimensional space, represented here in two-dimensions. Lines link similar protein sequences, with the connections shown here representing an E-value cut-off of $1e^{-10}$ of YSD1_22 mapped on all 20 groups of TTPs. Chi, YSD1, T5 and Lambda sequences (Source Data) are highlighted in pink. CLANS analysis using sequences from which the non-structural domain 3 (Big-1/Big-2) was deleted is similar but most connections between clusters are absent (cf. Data Source). **c**. Despite the considerable sequence-based diversity revealed by CLANS, there are close structure-based relationships for TTPs. Cartoon representation of the tail structure colored in a blue-red rainbow representation from N- to C-terminus for domain 1. Considerable similarity is evident to other TTPs, as well as the tube proteins from bacterial T6SS and pyocin structures (Supplementary Tables 6, 8). **d.** Cartoon representation of the oligomeric unit of the siphovirus tails and other tubes. The outer sheath is represented as a blue hexagon.

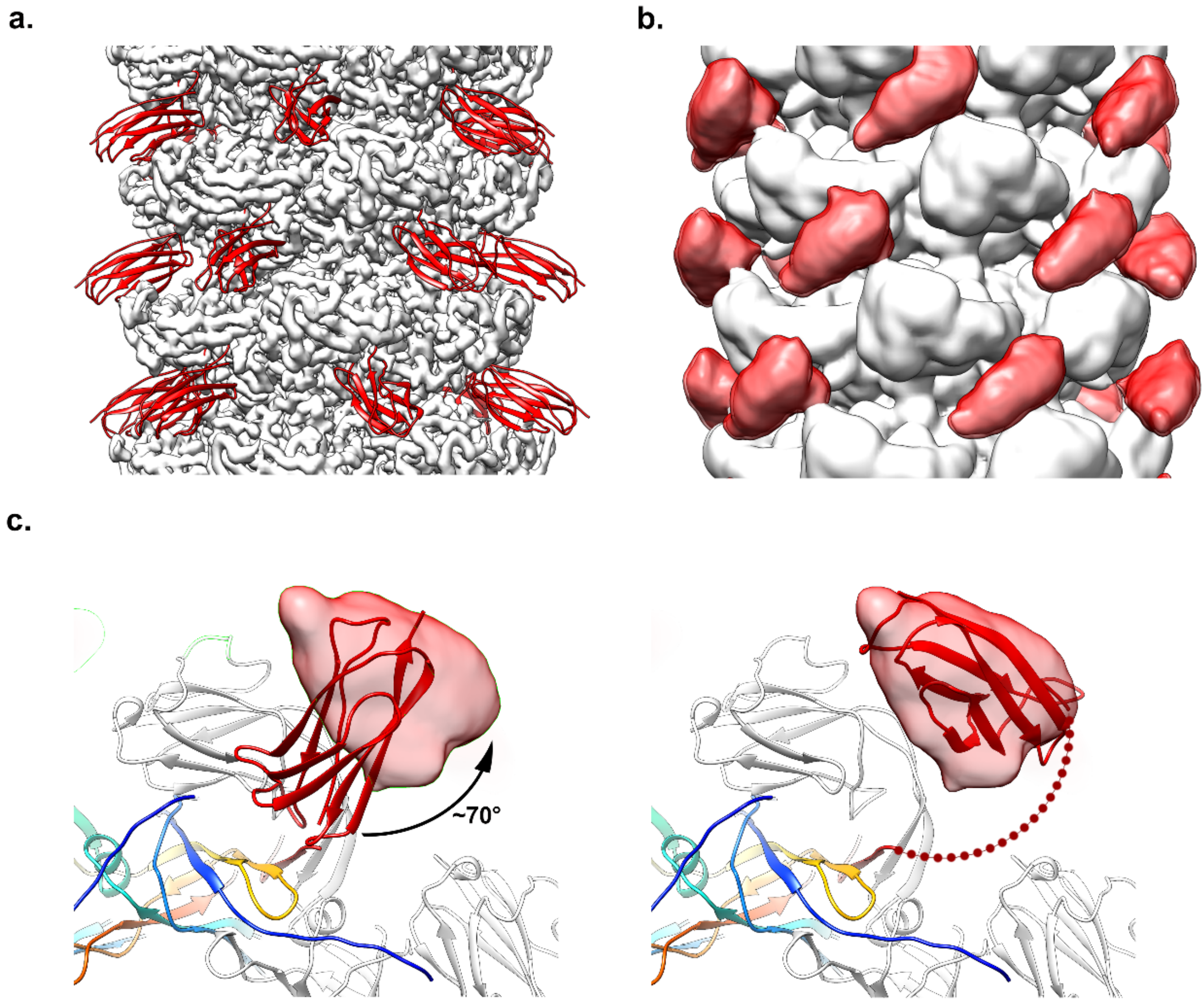

Supplementary Figure 8. Flexibility of domain 3 of the TTP in the EM reconstruction. a. Surface representation of the EM map (white) fitted with the YSD1_22 model atomic model and a model of domain 3 according to its corresponding position in the SAXS envelope (**Figure 4, Supplementary Figure 10**). **b.** When displayed at a lower threshold a blurred density is seen at the edges of the tube close to the predicted position of domain 3. **c.** Cartoon representation of domain 1 (rainbow), domain 2 (white), and domain 3 (red). A rotation of ~70° is required for an optimal fit in the extra density. There are is a stretch of 11 residues between domains 1 and 3, which may exist as a flexible linker between the two domains.

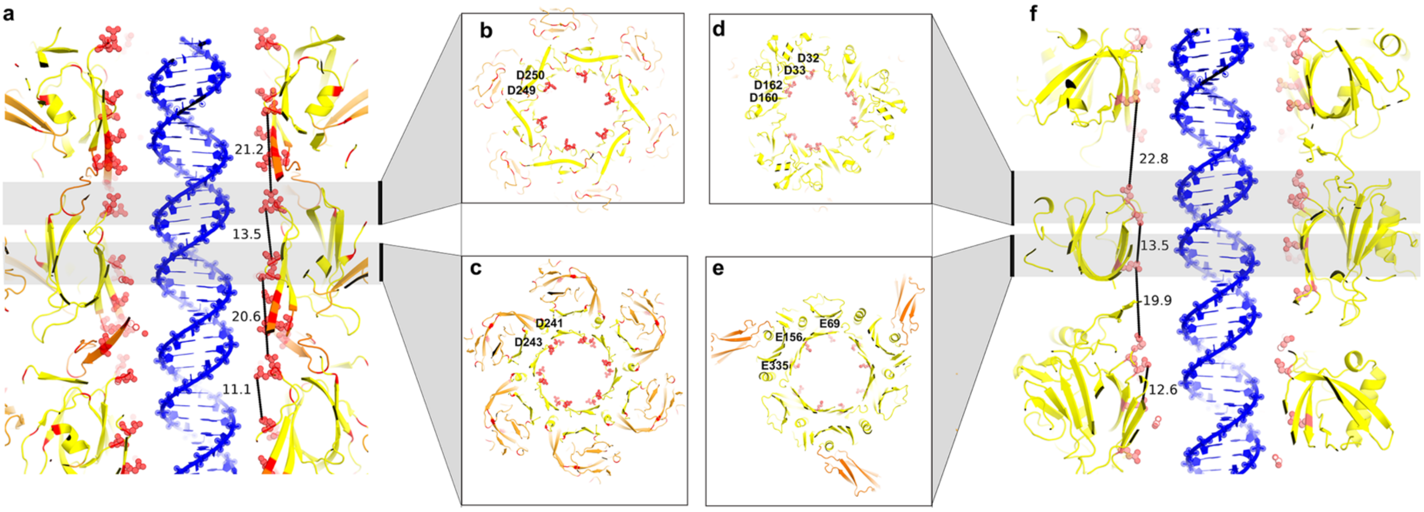

g

>lytic1-17temp12-13
MGMHLPNGSQIFIESSRGAEIAVTAATNAAASISDLTKGPVITVADASDGLAKGDYVIVTSSPWSKLLNRVLRVKTVTTAATTITLEGIDTTDTTKFPAGAFGVGTTGSVVKISSWTEIPCVQDVSTDGGEQQF
VTYQCLSDDREQQLPTYKSAVVLTYTFAHEYDNPIYPILRKLDESGAVTAIRMYVPKAASGKGEMRLWAGTVSFNEIPSTEVNEMETVSLAVTLKGRMSFLAADLVEALSKADLTDLPATKSVATGAALDLA
VVMKGGSAPYTYVWKKGSTAIPGKTASTFNISSVASGDAGVYTCEVTDAAGKTITSAACTVTVS

>lytic12
MANSVFNCSQVPAADVNATRLSIAKVCEPVSGTPWTVQQPNEISSYSADITKTQRTPISTDRSARKGTVTNVEVAPGFQTDITLDTFRYWGDGFLYSKWVGAGAIDIDVTSVDADSYNVATMGAALAAGTLVY
ATGFTLAANNGLKTVGASSTTTEIMVTGLAAEASPPAEARLYVVGHVAAAGDIAVNGNGQLTSTTLDFTTLGLVPGQYIYIDGFTQSVTSKMARVTTIDADTITLSNSEFTTEAGTDKTVRLFVSSFVRNVPVD
SADFLKTEYTMEARYNTTPVIYEYARAVAANQMTINAPLTEKMTMDLTFVAQDLSEPVETPLPGAGYSEFVANEAYNTVTNLNRVRLTGIDESGLSTYLKDVTVTINNNVSGENVLGVMGAAFTNIGNLEITM
DTETVMTDGSVLAAIRNNATVNFELAGVNGDGAFVVNIPAMTLGDGSKNLATGEKVKVTVSGTAHEEETVGYMIGFSLFPYLPTA

>lytic13 (Chi-like)
MNDNYQNNYVVGRGTVYFDRFQDGTNRKTGEMYFGNTPEFTINTDSETLDHYSSDHGMRVMDASVLLEASQGGTFTCDNINADNLALWFLGEVSNTTQTQQTDAKEVFNPIMRGRYYQLGTTDDNPTGVRGV
TNFQMVKADASIAISVGSGDITSIVGATVVNPAGNYEIDLEAGRIYIEPDSTDLAGNVQIAVQYDVDAQKRTLVIGKSNMVYGALRMISDNPVGLNKNYYFPKVSIAPDGDYALKGDDWQVMSFTFKAMQL
NNITQRVYIDIVEAAAAVDP

>lytic16
MMGFFKVKDVPSRRVVQYARVSGAGEGVVYIKDESVLGEPVDEMPFADKTGLALIADGILYEVPYLDDAGDVYFDLQPADTELKDG

>lytic4
MSLQLLRNTRIFVSTVKTGHNKTNTQEILVQDDISWGQDSNSTDITVNEAGPRPTRGSKRFNDSLNAAEWSFSTYILPYKDKTDGTDTNKQIVPDYMLWHALSSGKAINLEGDTGAHNNETNFMVNFKDNAY
HELAMLHIYILTDKAWSYIDSCQINQAEVNVDIEDIGRVTWSGNGNQLIPLDAAPFDPDTVGIDDETYMTIQGSYIKNKLTILKIKDMDSDKAYDIPITGGTFTINNNITYLTPNIMSRVNIPIGSFTGAF
ELTGSLTAYLNDKSLGSMELYKDLIKTLKVVNRFEIALILGGEYDDERPAAVLVAKQAHVNIPTIETDDVLGTSVEFKAIPTDLDTGDEGYLGFSSKYTKTTIANLIATGDGATAAPKK

>temp1-2-3-6
MSLPMKCEDAMPTPNPLAPVKGAGTTLWVYTGTGDAFANPLSDVDWLRLAKIKDLQPGEMTAESEDDTYLDDEDADWTATTQGQKSAGDTSFTLAWKPGESGQKDLVAWFDEGDVRAYKIKYPNGTVDV
FRGWVSSLGKTITAKEVITRTVKITNVGRPSLAEESGTAVIA

>temp23
MSNTHVKNIKLGACKVSFGGVDLGYTKGGVQVEVATETLKVTVDQLGQTVISELVQGRNITITAPLAESVLQNMVDLMPGSTLSEDDNSVTITSAQGVNLIDVAKELVLTPQDTTDYVLTIPKAATAGNFTMT
YQSDDVRVFSVQFSAYPDDEGVLGKMSGPK

>temp4-7
MKCRTIFRKTAVTVQPWSFRSRCNFFNRTHNPPRAGFLLSGGRMSALYEKSQLTKILISSAPATKETMDSLTFPGAGATFLDLSCTIKEIQFTGGQKQDIDVTTLCSTEQENINGLPAPSEISLSGNFYNNEP
AQDALRDAYDNDTTYGFQIIFPSGNGFKFLAEVRQHTWSSGTNGVVAATFSLRLKGKPVPIDSVLKLTTD

>temp4a
MSVLTQGTQLFVLVKGKVSEVECITAFSPGSNPADQIEDTCLSERFDRSYKRGLRTPGTASLTLNADPKNTSHIMLYNLSISDDEKDQDLTFAIGWSDGTASPTAAENGASGAVDGLVLPDSRTWFVFKGY
VSDFPFDFAANTVVSTSASIQRSGSAVWVPKVVTP

>temp5
MMACEAGAFTGRDVVVYYAIGCPEVQPTASAYQRLGMMRGKTVNAEWETADATADMSAAFTQENLVTYKNISFSGDGVTRKEDVYAQNALKRHVYNPPAETSNQPYVWFKIISPNDITEGPFMVTSWGDEAP
HDDVATWSIEASSAGQVDVRDVGAV

Supplementary Figure 9. Acidic clusters in tail tube proteins related to YSD1_22. a-f. Cut-away views of the tail tube of YSD1 (a-c) and T5 (d-f) phages with a double-stranded DNA segment represented at scale within the transit corridor. Acidic residues lining the inner surface of the tail tube are shown as red spheres and labelled. In panels b-e, longitudinal sections of the tail viewed from the phage head highlight the presence of dyads of acidic residues. g. Consensus sequences of the phage clusters defined by CLANS analysis in **Supplementary Figure 7**. Sequences have been truncated to include only the predicted equivalent of D1, and D2 where present. Acidic residues are shown in bold. Stretches of 2 or more acidic residues are colored in red and underlined, and D/ExD/E motifs in magenta where x is any residue.

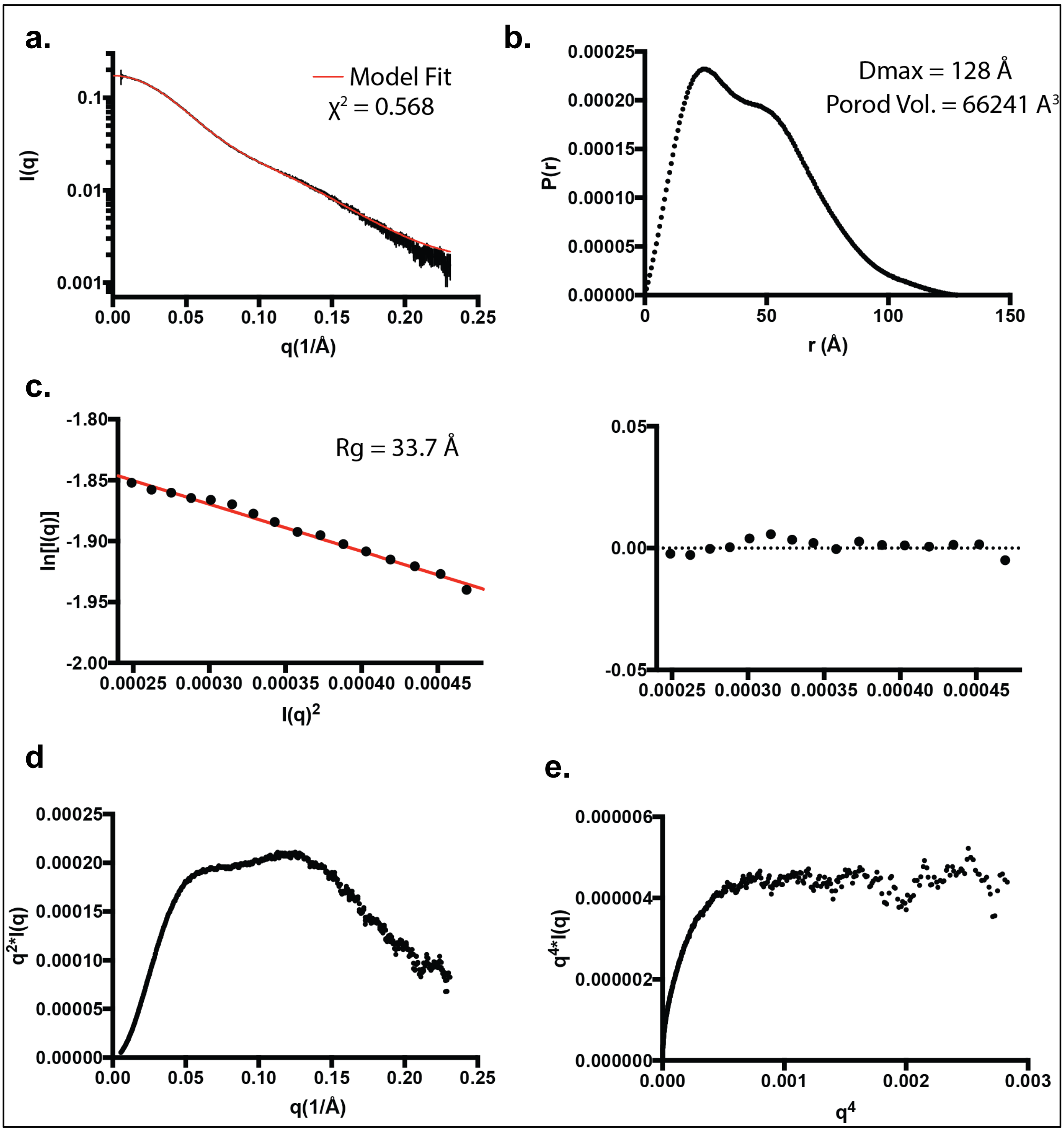

Supplementary Figure 10. Solution structure of the YSD1_22 monomer. a. Scattering angle vs. intensity plot for YSD1_22, the fit for the curve shown in red represents the YSD1_22 bead model generated with the program DAMMIF. **b**. P_r distribution showing that YSD1_22 has a maximum dimension of 128 Å, adopting a compact conformation in solution. **c.** A plot of the Guinier region of YSD1_22 SAXS showing the particle has a radius of gyration of 33.7 Å. **d.** Kratky plot supporting the multi-domain organisation of YSD1_22 with some evidence of inter-domain flexibility. **e.** Porod-Debye plot.

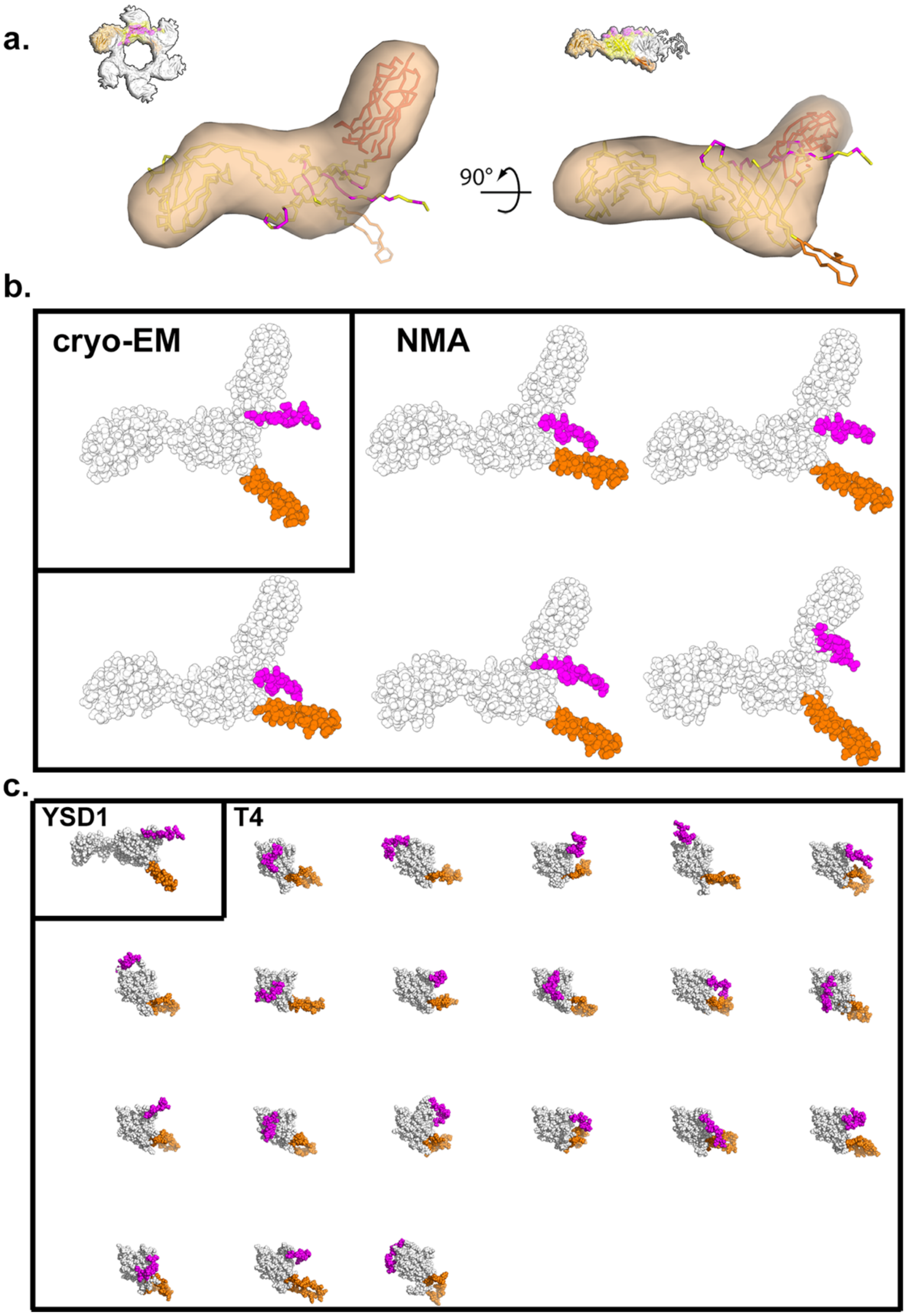

Supplementary Figure 11. Potential rearrangements in the monomeric form of YSD1_22. a. Orthogonal views of a molecular envelope of monomeric tail-tube protein derived from SAXS data (Supplementary Table 10). Insets indicate the orientation of each view with regard to the hexameric ring. The tail-tube protein structure from the assembled tail and a homology model for domain 3 are shown for comparison. In the ribbon representation, domains 1, 2 and 3 and the β-hairpin (β-HP) are shown in yellow, brown, cyan and orange, respectively. Residues involved in inter-ring contacts are shown in magenta. **b.** Comparison between the structure of YSD1_22 in the assembled tail and the top 5 models produced by normal mode analysis (NMA). All NMA models exhibited a comparable fit to the experimental SAXS data as described in Franke, et al. [11].(χ^2 = 0.51-0.56 vs static model χ^2 = 1.05). **c.** Comparison between the YSD1 tail protein and gpV, its homologue in phage lambda (pdb id 2k4q)[12]. The 20 deposited NMR models are represented with the same colour scheme and orientation as YSD1_22.

Supplementary Table 1. Cryo-EM data collection and reconstruction parameters

	YSD1 Capsid (EMDB-22183) (PDB 6XGQ)	YSD1 Tail (EMDB-22182) (PDB 6XGR)
**Data collection and processing**		
Magnification	105,000	105,000
Voltage (kV)	300	300
Electron exposure ($e^{–}$/Å^2)	27.24	27.24
Defocus range (μm)	0.5-2.5	0.5-2.5
Pixel size (Å)	0.67	0.67
Symmetry imposed	I	C6
Initial particle images (no.)	7,366	184,501
Final particle images (no.)	5,449	147,809
Map resolution (Å)	3.8	3.5
FSC threshold	0.143	0.143
Map resolution range (Å)	3.8-4.8	3.5-4.5
**Refinement**		
Initial model used (PDB code)	6XGP	N/A
Model resolution (Å)	3.8	3.5
FSC threshold	0.143	0.143
Map sharpening *B* factor (Å^2)	-134.0	-142.5
Model composition		
Non-hydrogen atoms	25,706	2,113
Protein residues	3318	275
Ligands	0	0
B factors (Å^2)		
Protein	76.3	71.65
Ligand	N/A	N/A
R.m.s. deviations		
Bond lengths (Å)	0.009	0.004
Bond angles (°)	0.932	0.668
Validation		
MolProbity score	1.79	1.41
Clashscore	5.94	2.92
Poor rotamers (%)	0	0
Ramachandran plot		
Favored (%)	93.25	95.2
Allowed (%)	6.75	4.8
Disallowed (%)	0	0

Supplementary Table 2. Crystallography data collection and refinement statistics on YSD1_17

	YSD1_17<br>(PDB 6XGP)
**Parameter**	
Space group	P1 2_1 1
Asymmetric unit	2 molecules
Cell dimensions	
a, b, c (Å)	54.40, 91.99, 89.03
α, β, γ (°)	90, 102.42, 90
Wavelength	0.9537
Resolution (Å)	46.01-2.6 (2.69-2.60)
Completeness (%)	99.6 (99.6)
I/σ<I>	8 (1.4)
CC1/2 (%)	98.3 (54.3)
Redundancy	2.0 (2.0)
R_{merge}	0.106 (0.734)
R_{meas}	0.149 (1.038)
R_{pim}	0.106 (0.734)
**Refinement Statistics**	
Resolution (Å)	30.83-2.6
No. of reflections	26349
R_{work}/R_{free}	0.219/0.258
No. of atoms	
Protein	5208
Water	46
Avg B factors	
Protein	44.6
Water	28.5
RMSD	
Bond length (Å)	0.01
Bond angle (°)	1.80
Validation	
MolProbity score (percentile)	100th
Clashscore	2.82
Poor rotamers (%)	2.77%
Ramachandran plot (%)	
Favored regions	98.0%
Allowed regions	1.7%
Outliers	0.3%

Values in parentheses are for highest resolution shell.

Supplementary Table 3. Structurally related proteins of the YSD1 MCP (mature capsid)

PDB	Z-score	RMSD	Alignment length	Number of residues	Sequence id. (%)	Virus	Family	MCP class	Protein
**3bqw**	22.1	3.6	270	347	22	-	-	-	Putative capsid protein of prophage
**5vf3**	13.7	3.6	257	456	6	T4	*Myo*	P22	Capsid vertex protein gp24 (isometric capsid)
**3bjq**	13.5	4.9	222	293	10	-	-	-	Phage-related protein
**1ohg**	13.3	3.5	243	280	6	HK97	*Sipho*	HK97	MCP (mature empty capsid)
**3j7x**	13.2	4	262	343	6	T7	*Podo*		MCP 10A (mature capsid)
**3j7w**	13.2	4.2	261	343	7	T7	*Podo*		MCP 10A (early-stage DNA packaging intermediate)
**5uu5**	13.1	3.8	254	430	7	P22	*Podo*	P22	MCP (mature capsid)
**5lii**	12.8	3.7	250	433	8	phi812	*Myo*		MCP (mature capsid)
**5wk1**	12.1	4.8	250	294	6	TW1	*Sipho*		MCP (mature capsid)
**5tjt**	11.8	4.2	245	292	9	T5	*Sipho*		Decoration protein pb10
**2gp1**	11.7	4.1	215	254	7	HK97	*Sipho*	HK97	MCP (Prohead II)
**3jb5**	10.7	4.4	248	300	5	PA6	*Sipho*		MCP (procapsid)
**5l35**	10.5	4	245	422	9	Sf6	*Podo*		MCP (mature capsid)
**2e0z**	10.3	4.1	201	228	12	-	-		PfV
**3dkt**	10.3	6.1	205	264	10	-	-		Maritimacin
**3qpr**	10.2	4.4	204	256	6	HK97	*Sipho*	HK97	MCP (Prohead I)
**4pt2**	10.1	5.3	223	277	9	-	-		Encapsulin protein
**6c21**	9.3	4	221	275	5	80alpha	*Sipho*	λ/χ	MCP (mature capsid)
**3j7v**	9.3	5.4	226	296	8	T7	*Podo*		MCP 10A (procapsid)
**1yue**	9.3	6	219	390	10	T4	*Myo*	P-22	Capsid vertex protein gp24 (prolate capsid)
**6b0x**	8.7	5.7	210	284	6	80alpha	*Sipho*	λ/χ	MCP (procapsid)
**2xvr**	8.5	3.9	215	246	6	T7	*Podo*		MCP 10A (mature empty capsid)
**3j4u**	6.9	3.9	181	327	5	BPP-1	*Podo*	BPP-1	MCP (mature capsid)
**3j40**	6.4	3.9	175	335	6	Epsilon15	*Podo*	BPP-1	MCP gp10 (mature capsid)

Generated using the DALI server[13]. The RMSD is reported for all aligned Cα as defined in the "Alignment length" column. Redundant structures have been omitted.

Supplementary Table 4. Structurally related proteins of the YSD1 MCP (monomer).

PDB	Z-score	RMSD	Alignment length	Number of residues	Sequence id. (%)	Virus	Family	MCP class	Protein
**3bqw**	31.4	2.6	313	347	25	-	-	-	Putative capsid protein of prophage
**3bjq**	16	3.6	242	296	11	-	-	-	Phage-related protein
**2gp1**	12.2	3.6	211	254	7	HK97	*Sipho*	HK97	MCP (Prohead II)
**5vf3**	12	4.8	220	456	6	T4	*Myo*	P22	Capsid vertex protein gp24 (isometric capsid)
**5uu5**	11.7	4	224	430	9	P22	*Podo*	P22	MCP (mature capsid)
**2e0z**	11.5	3.8	208	228	13	-	-	-	PfV
**1ohg**	11.5	4.1	203	280	7	HK97	*Sipho*	HK97	MCP (mature empty capsid)
**3j7w**	11.1	4.5	226	343	7	T7	*Podo*		MCP 10A (early-stage DNA packaging intermediate)
**3dkt**	11.1	4.7	214	264	10	-	-	-	Maritimacin
**4pt2**	11	4	223	277	10	-	-	-	Encapsulin protein
**3j7x**	10.7	4.8	221	343	8	T7	*Podo*		MCP 10A (mature capsid)
**3qpr**	10.6	4	213	256	7	HK97	*Sipho*	HK97	MCP (Prohead I)
**5lii**	10.4	3.6	207	433	11	phi812	*Myo*		MCP (mature capsid)
**1yue**	10.2	4.8	217	390	9	T4	*Myo*	P22	Capsid vertex protein gp24 (prolate capsid)
**3j7v**	9.9	4.5	229	298	8	T7	*Podo*		MCP 10A (procapsid)
**5wk1**	9.8	4.4	220	294	7	TW1	*Sipho*		MCP (mature capsid)
**5tjt**	9.2	4.9	201	292	9	T5	*Sipho*		Decoration protein pb10
**5l35**	9.1	4.7	201	422	9	Sf6	*Sipho*		MCP (mature capsid)
**3jb5**	8.9	4.8	207	300	7	PA6	*Sipho*		MCP (procapsid)
**6b0x**	8.8	4.6	215	284	8	80alpha	*Sipho*	λ/χ	MCP (procapsid)
**6c21**	8.3	4.3	190	275	7	80alpha	*Sipho*	λ/χ	MCP (mature capsid)
**2xvr**	7.6	3.9	189	246	6	T7	*Podo*		MCP 10A (mature empty capsid)
**3j40**	3.8	5	146	335	5	Epsilon15	*Podo*	BPP-1	MCP gp10 (mature capsid)

Generated using the DALI server[13]. The RMSD is reported for all aligned Cα as defined in the "Alignment length" column. Redundant structures have been omitted.

Supplementary Table 5. Structurally related proteins to YSD1_16

PDB	Z-score	RMSD	Alignment length	Number of residues	Sequence id. (%)	Virus	Protein
**1td4**	12.8	1.7	95	102	25	P21	SHP
**1c5e**	11.9	1.7	88	95	21	Lambda	gpD
**6bl5**	6.1	2.7	86	129	9	P74-26	P87
**5wk1**	5.2	3.2	85	148	16	TW1	TW1_056
**3suc**	4.8	11.4	80	767	10	phi29	Preneck appendage protein

Generated using the DALI server[13]. The RMSD is reported for all aligned Cα as defined in the "Alignment length" column. Redundant structures have been omitted.

Supplementary Table 6. Structurally related proteins of the YSD1 TTP domain 1

PDB	Z-score	RMSD	Alignment length	Number of residues	Sequence id. (%)	Organism	Description
**4hkh**	9.9	2.9	113	137	5	*Escherichia coli*	Putative T6SS
**3he1**	9.8	2.7	107	143	7	*Pseudomonas aeruginosa*	T6SS Hcp3 protein
**6a2v**	9.5	3	113	148	9	*Campylobacter jejuni*	T6SS Hcp1
**6bdc**	9.5	3.2	112	123	5	*Flavobacterium johnsoniae*	T6SS Hcp1
**5xeu**	9.1	2.7	101	129	12	*Salmonella typhimurium*	T6SS Hcp2
**5mxn**	9	2.9	120	170	11	*Vibrio cholerae*	Non-contractile T6SS
**5w5e**	8.6	3	128	167	8	*Pseudomonas aeruginosa*	Pyocin FIIR2 protein
**5ngj**	8.6	3.6	105	432	8	T5 phage	pb6 TTP
**4ksr**	8.5	2.8	115	521	10	*Vibrio cholerae*	T2SS protein E
**5w5f**	7.9	3.2	129	163	9	T4 phage	gp19 TTP

Generated using the DALI server[13]. The RMSD is reported for all aligned Cα as defined in the “Alignment length” column. Redundant structures have been omitted.

Supplementary Table 7. PISA analysis of YSD1_22 in the structural context of the tail-tube

Interface	Buried Surface (Å^2)	Free energy ($\Delta^i G$ kcal/mol)	Hydrogen bonds	Salt bridges
**Within ring**	2664.9	-19.0	32	11
**Within ring (domain 1 with n-1)**	1665.9	-16.6	19	2
**Within ring (domain 2 with n-1)**	949.8	-3.0	12	9
**Within ring (domain 2 with n-2)**	124.9	0.4	1	0
**Interring**	876.2	-4.8	9	4
**Inter-ring (without β-hairpin)**	61.5	0.4	1	0
**Inter-ring (without β-hairpin, without N-terminus)**	0	N/A	0	0

Supplementary Table 8. Structurally related proteins of the YSD1 TTP domain 2

PDB	Z-score	RMSD	Alignment length	Number of residues	Sequence id. (%)	Organism	Description
**2w38**	6.2	5.9	82	432	12	*Pseudomonas aeruginosa*	Sialidase-like protein
**4mbo**	5.5	3.8	88	308	15	*Streptococcus agalactiae*	Serine-rich repeat adhesion glycoprotein (SRR1)
**2zuw**	5	4.8	85	745	11	*Bifidobacterium longum*	Lacto-N-biose phosphorylase
**5cfa**	4.8	3.3	83	326	10	*Staphylococcus aureus*	Bone sialoprotein-binding protein
**3is1**	4.8	3.7	87	393	9	*Staphylococcus saprophyticus*	Uro-adherence factor A
**4igb**	4.8	4	78	426	12	*Streptococcus gordonii*	Adhesin Sgo0707
**5hx2**	4.8	6	81	647	7	T4 phage	T4 baseplate wedge protein gp7
**5jq6**	4.7	4	93	320	10	*Staphylococcus aureus*	Clumping factor A
**4zqt**	4.6	2.9	76	889	7	*Plasmodium falciparum*	M1 family aminopeptidase
**2y1v**	4.6	3.1	80	604	4	*Streptococcus pneumoniae*	Pilus protein
**1r19**	4.6	4	78	286	9	*Staphylococcus epidermidis*	Fibrinogen-binding protein SdrG
**4b60**	4.5	3.4	84	299	12	*Staphylococcus aureus*	Fibronectin-binding protein A
**6n38**	4.5	4.8	82	532	9	*Escherichia coli*	T6SS Baseplate TssF protein

Generated using the DALI server[13]. The RMSD is reported for all aligned Cα as defined in the “Alignment length” column. Redundant structures have been omitted.

Supplementary Table 9. Curated list of *Siphoviridae* genomes used for phylogenetic tree analysis

Cluster	Phage Name	Accession Number
**Lytic1**	T1	AY216660
	Shfl1	NC_015456
	ADB-2	JX912252
	Rogue1	JQ182736
	JK06	DQ121662
	ΦJLA23	KC333879
	ΦKP26	KC579452
	ΦEB49	JF770475
	ACG-M12	NC_019404
	F20	JN672684
	TLS	AY308796
	ESP2949	JF912400
	pSf-1	KC710998
**Lytic4**	T5	AY543070
	EPS7	CP000917
	SPC35	HQ406778
	AKFV33	NC_017969
	ΦR201	HE956708
	My1	JX195166
**Lytic12**	9NA	KJ802832
	FSL_SP-062	KC139632
	FSL_SP-069	KC139649
**Lytic13**	Chi	JX094499
	FSL_SP-030	KC139519
	FSL_SP-039	KC139514
	FSL_SP-088	KC139512
	FSL_SP-124	KC139515
	iEPS5	KC677662
	SPN19	JN871591
	Enc34	JQ340774
	RedJac	NC_018832
	YSD1	LR026998
**Lytic16**	SETP3	EF177456
	AG11	JX297445
	SETP7	KF562865
	SETP13	KF562864
	Ent1	HE775250
	SE2	JQ007353
	SSe3 (KS7)	AY730274
	wskl3	JX202565
	FSL_SP-101	KC139511
	Jersey	KF148055
	K1-dep(4) / (K1G)	GU196277
	K1-dep(1) / (K1H)	GU196278
	K1-ind(1)	GU196279
	K1-ind(2)	GU196280
	K1-ind(3)	GU196281
	FSL_SP-031	KC139518
	Eta	KC460990
**Lytic17**	SO-1	GQ502199
	EP23	JN984867
	EEP (SSL-2009a)	FJ750948
	HK578	JQ086375
	JL1	JX865427
	eiAU	KF772233
	eiAU-183	KF772234
**Lytic26**	E1	AM491472
**Lytic31**	ΦEaH1	KF623294
**Lytic32**	9g	KJ419279

**Temp1**	Lambda	J02459
	HK630	JQ086376
	HK629	JQ182735
**Temp2**	Φ80	JX871397
	HK225	JQ086371
	mEp237	JQ182730
**Temp3**	N15	AF064539
	PY54	AJ564013
	ΦKO2	AY374448
**Temp4**	HK97	AF069529
	HK022	AF069308
	HK75	HM173637
	HK106	JQ086369
	HK140	JQ086370
	HK446	JQ086372
	HK542	JQ086373
	HK544	JQ086374
	HK633	JQ086377
	mEpX1	JQ182727
	mEpX2	JQ182726
	mEp234	JQ182732
	mEp235	JQ182731
	mEp390	JQ182729
	ENT39118	HQ201307
**Temp5**	ES18	AY736146
	Oslo	NC_018279
	SPN3UB	JQ288021
**Temp6**	gifsy-2	NC_010393
	gifsy-1	NC_010392
	Fels-1	NC_010391
	mEp043	JQ182734
	mEp213	JQ182733
	CP-1639	AJ304858
	CTD-IΦ	AB285204
	mEp460	JQ182728
	FSL_SP-016	KC139516
**Temp7**	BP-4795	AJ556162
	2851	FM180578
	Stx2-1717	NC_011357
	YYZ-2008	NC_011356
**Temp12**	HK639	NC_016158
**Temp13**	ΦES15	JQ780327
**Temp23**	SSU5	JQ965645

Supplementary Table 10. SAXS data collection and analysis

	YSD1_22
**Sample Parameters**	
**Organism**	YSD1 phage
**Source**	*E. coli* (recombinant expression)
**Genbank sequence ID (residues in construct)**	EDG6582788 (1-381)
**Extinction coefficient [A280, 0.1%(w/v)]**	0.829
**M from chemical composition (Da)**	41430
SEC–SAXS column, 5 150 mm Superdex S200	
**Loading concentration (mg.ml^{1})**	10.0
**Injection volume (µl)**	50
**Flow rate (ml.min^{1})**	0.4
**Solvent (solvent blanks taken from SEC flowthrough prior to elution of protein)**	50 mM Tris, 100 mM NaCl, 5% Glycerol, 0.1% NaN_3 pH 7.9
**Data-collection parameters**	
**Instrument**	Australian Synchrotron SAXS/WAXS beamline with Dectris PILATUS 1M detector
**Wavelength (Å)**	1.0322
**Beam size (µm)**	250 × 130
**Camera length (m)**	2.683
**q range (Å^{-1})**	0.00663-0.3104
**Sample configuration**	Sample configuration SEC–SAXS with sheath-flow cell mm
**Temperature (K)**	295
**Structural Parameters**	
Guinier Analysis	
**I(0) (cm^{-1})**	0.17 +/-0.00038
**R_g (Å)**	33.90 +/-0.12
**Coefficient of correlation, R^2**	1.00
P(r) Analysis	
**I(0) (cm^{-1})**	0.17 +/-0.00036
**R_g (Å)**	33.93 +/-0.07
**D_{max} (Å)**	103.48
**q range (Å^{-1})**	0.007-0.241
**χ^2 (total estimate from GNOM)**	0.777 (0.82)
**Porod volume estimate (Å^3)**	53516
**Dry volume calculated from sequence (Å^3)**	48843
**Molecular-mass determination**	
**Molecular mass (*M*) [from Bayesian Inference] (Da)**	44700
**Calculated monomeric *M* from sequence (Da)**	41430
**Ratio *M* calculated to expected**	1.08

**Shape Model Fitting Results**	
**DAMMIF**	
**q range (Å^{-1}) for fitting**	0.007-0.241
**Symmetry, anisotropy assumptions**	P1, none
**χ^2 range**	0.52-63
**Constant adjustment to intensities**	Skipped, unable to determine
***M* estimate as 0.5 volume of models (Da) (ratio to expected)**	37941 (0.78)
**Atomistic Modelling**	
CRYSOL/SREFLEX	
**Atomic Model**	YSD1_22 Cryo-EM structure + Domain 3 model (this study)
**q range (Å^{-1}) for all modelling**	0.007-0.241
Constant subtraction allowed	
**χ^2, P-value**	1.05, 0.01
**Predicted R_g (Å)**	34.20
**Vol (Å), Ra (Å), Dro (eÅ^3)**	44154, 1.44, 0.003
**Top Normal Modes Explored**	16.0
**χ^2 Range for NMA models**	0.51-0.56
**Software Employed**	
**SAXS data reduction**	*I(q)* versus *q* using ScatterBrain 2.82
**Extinction coefficient estimate**	ProtParam
**Basic analyses: Guinier, P(r), V_p**	PRIMUSqt from ATSAS 2.8.1
**Shape/bead modelling**	DAMMIF from PRIMUSqt in ATSAS 2.8.1
**Atomic structure modelling**	CRYSOL/SREFLEX from PRIMUSqt in ATSAS 2.8.1
**SASBDB IDs for data and model**	SAS2352

Supplementary References

1 Berg, H. C. & Anderson, R. A. Bacteria swim by rotating their flagellar filaments. *Nature* **245**, 380-382, doi:10.1038/245380a0 (1973).

2 Katsamba, P. & Lauga, E. Hydrodynamics of bacteriophage migration along bacterial flagella. *Physical Review Fluids* **4**, doi:10.1103/PhysRevFluids.4.013101 (2019).

3 Grose, J. H. & Casjens, S. R. Understanding the enormous diversity of bacteriophages: The tailed phages that infect the bacterial family Enterobacteriaceae. *Virology* **468-470**, 421-443, doi:10.1016/j.virol.2014.08.024 (2014).

4 Pettersen, E. F. *et al.* UCSF Chimera--a visualization system for exploratory research and analysis. *J. Comput. Chem.* **25**, 1605-1612, doi:10.1002/jcc.20084 (2004).

5 Notredame, C., Higgins, D. G. & Heringa, J. T-Coffee: A novel method for fast and accurate multiple sequence alignment. *J. Mol. Biol.* **302**, 205-217, doi:10.1006/jmbi.2000.4042 (2000).

6 Robert, X. & Gouet, P. Deciphering key features in protein structures with the new ENDscript server. *Nucleic Acids Res.* **42**, W320-324, doi:10.1093/nar/gku316 (2014).

7 Sievers, F. & Higgins, D. G. Clustal Omega for making accurate alignments of many protein sequences. *Protein science : a publication of the Protein Society* **27**, 135-145, doi:10.1002/pro.3290 (2018).

8 Desfosses, A., Ciuffa, R., Gutsche, I. & Sachse, C. SPRING - an image processing package for single-particle based helical reconstruction from electron cryomicrographs. *J. Struct. Biol.* **185**, 15-26, doi:10.1016/j.jsb.2013.11.003 (2014).

9 He, S. & Scheres, S. H. W. Helical reconstruction in RELION. *J. Struct. Biol.* **198**, 163-176, doi:10.1016/j.jsb.2017.02.003 (2017).

10 Frickey, T. & Lupas, A. CLANS: a Java application for visualizing protein families based on pairwise similarity. *Bioinformatics* **20**, 3702-3704, doi:10.1093/bioinformatics/bth444 (2004).

11 Franke, D. *et al.* ATSAS 2.8: a comprehensive data analysis suite for small-angle scattering from macromolecular solutions. *J Appl Crystallogr* **50**, 1212-1225, doi:10.1107/S1600576717007786 (2017).

12 Pell, L. G., Kanelis, V., Donaldson, L. W., Howell, P. L. & Davidson, A. R. The phage lambda major tail protein structure reveals a common evolution for long-tailed phages and the type VI bacterial secretion system. *Proc. Natl. Acad. Sci. U. S. A.* **106**, 4160-4165, doi:10.1073/pnas.0900044106 (2009).

13 Holm, L. & Laakso, L. M. Dali server update. *Nucleic Acids Res.* **44**, W351-355, doi:10.1093/nar/gkw357 (2016).
