# Supplementary material for: The architecture and stabilisation of flagellotropic tailed bacteriophages
Source: Nat Commun. 2020 Jul 27;11:3748. doi: 10.1038/s41467-020-17505-w (PMC7385642; doi:10.1038/s41467-020-17505-w)
Supplement: Supplementary file 4 — Reporting Summary [file 41467_2020_17505_MOESM4_ESM.pdf]

nature research

Corresponding author(s): Fasseli Coulibaly

Last updated by author(s): Jun 22, 2020

Reporting Summary

Nature Research wishes to improve the reproducibility of the work that we publish. This form provides structure for consistency and transparency in reporting. For further information on Nature Research policies, see Authors & Referees and the Editorial Policy Checklist .

Statistics

For all statistical analyses, confirm that the following items are present in the figure legend, table legend, main text, or Methods section.

n/a	Confirmed	
x	☐	The exact sample size (*n*) for each experimental group/condition, given as a discrete number and unit of measurement
x	☐	A statement on whether measurements were taken from distinct samples or whether the same sample was measured repeatedly
x	☐	The statistical test(s) used AND whether they are one- or two-sided<br>*Only common tests should be described solely by name; describe more complex techniques in the Methods section.*
x	☐	A description of all covariates tested
x	☐	A description of any assumptions or corrections, such as tests of normality and adjustment for multiple comparisons
x	☐	A full description of the statistical parameters including central tendency (e.g. means) or other basic estimates (e.g. regression coefficient) AND variation (e.g. standard deviation) or associated estimates of uncertainty (e.g. confidence intervals)
x	☐	For null hypothesis testing, the test statistic (e.g. *F*, *t*, *r*) with confidence intervals, effect sizes, degrees of freedom and *P* value noted<br>*Give P values as exact values whenever suitable.*
x	☐	For Bayesian analysis, information on the choice of priors and Markov chain Monte Carlo settings
x	☐	For hierarchical and complex designs, identification of the appropriate level for tests and full reporting of outcomes
x	☐	Estimates of effect sizes (e.g. Cohen's *d*, Pearson's *r*), indicating how they were calculated

Our web collection on statistics for biologists contains articles on many of the points above.

Software and code

Policy information about availability of computer code

Data collection	SerialEM 3.6
Data analysis	XDS (v. 20151015), Aimless (v. 0.7.3), CCP4 7.0, PHASER 2.6.0, BUSTER 2.10.3, COOT 0.8.9.1, CHIMERA 1.13.1, PyMOL 2.0.6, MotionCor2 1.1.0, CTFFIND 4.1.8, RELION 2.1, EMAN 2.12, SPRING 0.86.1661, ROSETTA 1.16, PHENIX 1.13, APBS 3.7, ATSAS 3.0.2

For manuscripts utilizing custom algorithms or software that are central to the research but not yet described in published literature, software must be made available to editors/reviewers. We strongly encourage code deposition in a community repository (e.g. GitHub). See the Nature Research guidelines for submitting code & software for further information.

Data

Policy information about availability of data

All manuscripts must include a data availability statement. This statement should provide the following information, where applicable:

- Accession codes, unique identifiers, or web links for publicly available datasets
- A list of figures that have associated raw data
- A description of any restrictions on data availability

The coordinates and diffraction data of the major capsid protein crystal structure have been deposited in the Protein Data Bank (YSD1_17; PDB ID: 6XGP). The coordinates for the capsid (YSD1_16 and YSD1_17; PDB ID: 6XGQ) and tail-tube (YSD1_22; PDB ID: 6XGR) from the cryo-EM structure of the mature YSD1 phage have been deposited in the Protein Data Bank. The icosahedral reconstruction of the capsid (EMDB ID: 22183) and helical reconstruction of the tail (EMDB ID: 22182) have been deposited to the Electron Microscopy Bank. SAXS scatter and envelope data for YSD1_22 have been deposited in the Small angle scattering data bank (SASDB ID: SASDJM2). Source data are provided with this paper.

Field-specific reporting

Please select the one below that is the best fit for your research. If you are not sure, read the appropriate sections before making your selection.

[x] Life sciences [ ] Behavioural & social sciences [ ] Ecological, evolutionary & environmental sciences

For a reference copy of the document with all sections, see nature.com/documents/nr-reporting-summary-flat.pdf

Life sciences study design

All studies must disclose on these points even when the disclosure is negative.

Sample size	Sample size is not relevant to structure determination experiments. Relevant statistics for data completeness is provided in the crystallography section and image/particle numbers under the cryo-EM section.
Data exclusions	Outliers in diffraction data were removed during data reduction using XDS with default parameters as preset in XDS and standard practice. Cryo-EM images of poor quality were removed based on visual inspection and assessment of the contrast transfer function.
Replication	Structure determination by X-ray crystallography and cryo-EM and the SAXS analysis were validated internally using accepted quality criteria as described in the manuscript and thus were not replicated. Size-exclusion chromatography was repeated at least 3 times for each sample using two independent purifications for YSD1_22 and its delta N deletion mutant, or one purification for the delta-HP and double mutant. Imaging of the virus particles by negative-stain EM was performed on samples from three independent purifications and cryo-EM on one purification (5 imaging sessions).
Randomization	Randomization is not relevant to structure determination experiments. Relevant statistics are provided in the crystallography, SAXS and cryo-EM sections.
Blinding	Structure determination was performed according to standard practice. With regard to blinding, the Rfree factor was used to provide an unbiased assessment of the crystal structure quality. The Fourier Shell Correlation between two half-dataset was used to provide a "gold-standard" estimate of the resolution of the cryo-EM structures.

Reporting for specific materials, systems and methods

We require information from authors about some types of materials, experimental systems and methods used in many studies. Here, indicate whether each material, system or method listed is relevant to your study. If you are not sure if a list item applies to your research, read the appropriate section before selecting a response.

Materials & experimental systems

n/a	Involved in the study
[x]	[ ] Antibodies
[x]	[ ] Eukaryotic cell lines
[x]	[ ] Palaeontology
[x]	[ ] Animals and other organisms
[x]	[ ] Human research participants
[x]	[ ] Clinical data

Methods

n/a	Involved in the study
[x]	[ ] ChIP-seq
[x]	[ ] Flow cytometry
[x]	[ ] MRI-based neuroimaging
