# Supplementary material for: The architecture and stabilisation of flagellotropic tailed bacteriophages
Source: Nat Commun. 2020 Jul 27;11:3748. doi: 10.1038/s41467-020-17505-w (PMC7385642; doi:10.1038/s41467-020-17505-w)
Supplement: Supplementary file 5 — Description of Additional Supplementary Files [file 41467_2020_17505_MOESM5_ESM.pdf]

## Description of Additional Supplementary Files

**File Name**: Supplementary Movie 1

**Description:** Acidic dyads in the YSD1 tail tube match features in DNA, which may facilitate genome exit. Representation of the YSD1 tail modelled with a double-stranded DNA molecule in its B form. The movie relates to Figure 7 and Supplementary Figure 9. Domains 1 and 2 of the tail tube protein are shown in yellow and orange, respectively. DNA is shown in blue. The side chains of conserved acidic residues forming a helical track on the inner surface of the tube are shown as red spheres. The repeats match features in double-stranded DNA, which may facilitate genome translocation to infect the bacterial target.
